# Evaluation of a university hospital trauma team activation protocol

**DOI:** 10.1186/1757-7241-19-18

**Published:** 2011-03-28

**Authors:** Trond Dehli, Knut Fredriksen, Svein A Osbakk, Kristian Bartnes

**Affiliations:** 1 Department of Gastrointestinal Surgery, University Hospital of North Norway Tromsø, 9038 Tromsø, Norway; 2 Division of Emergency Medical Services, University Hospital of North Norway Tromsø, 9038 Tromsø, Norway; 3 Anaesthesia and Critical Care Research Group, Department of Clinical Medicine, Faculty of Health Sciences, University of Tromsø, 9037 Tromsø, Norway; 4 Department of Cardiothoracic and Vascular Surgery, University Hospital of North Norway Tromsø, 9038 Tromsø, Norway

## Abstract

**Background:**

Admission with a multidisciplinary trauma team may be vital for the severely injured patient, as this facilitates rapid diagnosis and treatment. On the other hand, patients with minor injuries do not need the trauma team for adequate care. Correct triage is important for optimal resource utilization. The aim of the study was to evaluate our criteria for activating the trauma team, and identify suboptimal criteria that might be changed in the interest of precision.

**Methods:**

The study is an observational, retrospective cohort-study. All patients admitted with the trauma team (n = 382), all severely injured (Injury Severity Score (ISS) >15) (n = 161), and all undergoing an emergency procedure aimed at counteracting compromised airways, respiration or circulation at our hospital (n = 142) during 2006-2007 were included. Data were recorded from the admission records and the electronic patient records. The trauma team activation protocol was evaluated against the occurrence of severe injury and the occurrence of emergency procedures.

**Results:**

A total of 441 patients were included. The overtriage was 71% and undertriage 32% when evaluating against ISS >15 as the standard of reference. When occurrence of emergency procedures was held as the standard of standard of reference, the over- and undertriage was 71% and 21%, respectively. Mechanism of injury-criteria for trauma team activation contributed the most to overtriage. The emergency procedures performed were mostly endotracheal intubation and external fixation of fractures. Less than 3% needed haemostatic laparotomy or thoracotomy. Approximately 2/3 of the overtriage represented isolated head or cervical spine injuries, and/or interhospital transfers.

**Conclusions:**

The over- and undertriage of our protocol are both too high. To decrease overtriage we suggest omissions and modifications of some of the criteria. To decrease undertriage, transferred patients and patients with head injuries should be more thoroughly assessed against the trauma team activation criteria.

## Background

Multidisciplinary trauma teams reduce mortality and have become an important part of modern trauma care [1]. Protocols for trauma team activation (TTA) are mainly based on prehospital information and aim at ensuring that the severely injured receive multidisciplinary care immediately upon admission, while limiting the waste of resources caused by excessive team mobilizations. TTA guidelines are widely implemented throughout Scandinavia [2]. Although they vary somewhat, the TTAs of most Scandinavian trauma centers comply with the recommendations of the American College of Surgery - Committee On Trauma (ACS-COT) [3-8] and rely on parameters of physiologic compromise, anatomic damage, and mechanism of injury (MOI). A substantial overtriage (activation of the trauma team despite minor or moderate injury) is common and may reach 70%, mostly reflecting the limited precision of criteria relating to MOI [5-7]. Overtriage is mainly a resource problem, as assembly of the multidisciplinary trauma team diverts personnel from other important activities in the hospital. Undertriage delays diagnosis and treatment of severely injured patients, and may compromise clinical outcome and increase trauma mortality [8]. ACS-COT suggests that an overtriage as high as 50% is acceptable if necessary to minimize undertriage [3].

Triage criteria should be adapted to the local case-load and injury pattern, which may vary considerably between geographical regions. The predictive properties of triage criteria depend on the prevalence and spectrum of severe injuries. Typical for most Scandinavian hospitals receiving trauma patients is a predominance of blunt over penetrating injuries [9,10]. Furthermore, the frequency of severe polytrauma admissions is low [11]. The present study was initiated as we frequently observed TTA for patients with an Injury Severity Score (ISS) <15 and without a need for emergency procedures to stabilize airway, respiration or circulation. The aim was to establish the predictive properties of our TTA protocol and its individual criteria in an effort to improve the protocol's precision.

## Material and methods

### Study design

The study is a retrospective observational cohort study.

### Clinical setting

The study was conducted at the University Hospital of North Norway Tromsø (UNN), which serves 120.000 inhabitants as a local hospital. It is also the regional trauma center for North Norway, including the arctic Svalbard archipelago. The region has 468.000 inhabitants, the mainland part covers an area of 107 000 km2, and spans the same length as the British Isles from south to north. There are 11 hospitals receiving polytrauma patients, eight of these have less than 50 TTA a year [12]. Those patients in need of transfer to the regional trauma centre, are transported by the well-developed national air ambulance system [13]. Typically, transport times range between 1 - 4 hrs, frequently prolonged due to difficult weather conditions. The trauma team in UNN consists of a general surgeon (team leader, senior registrar), orthopedic surgeon (registrar), anesthesiologist (senior registrar), nurse anesthetist, radiologist (registrar), radiographer, biochemist, nurse, and porter. The team is supplemented according to demand, most frequently with a neurosurgeon.

### Subjects

Trauma patients were identified from admission records of the UNN, where all referred patients were assessed according to receiving department, diagnosis at referral, activation of the trauma team, referring institution, emergency procedures and Injury Severity Score (ISS) to decide on inclusion or not. All patients received by the trauma team and all patients with ISS >15 or undergoing an emergency procedure (for stabilization of compromised airways, respiration or circulation) admitted during January 1st 2006 until December 31st 2007 were included [14,15]. Because transport delays are common, we included transfers from other hospitals up to 48 hours after the time of injury. Patients with burns are included, patients with asphyxia from strangulation or drowning as the only injury were excluded.

### Methods and data

The Emergency Medical Dispatch and Coordination Centre (EMDC) of the UNN activates the trauma team when prehospital information meets at least one of our TTA protocol criteria. As a consequence, not every criterion is checked for every patient, as this is not necessary for the decision to mobilize the team. We recorded all criteria documented by the EMDC, and also searched the admission note in the patient record for criteria known before arrival of the patient. Diagnoses, treatment, and outcomes were collected from the patient records of the hospital, including the records of the EMDC and the prehospital services. ISS calculations were based on a single surgeon's Abbreviated Injury Scale (AIS) scoring which was performed twice, several months apart [15]. A third scoring was performed if there were inconsistencies between these assessments. As emergency procedures we recorded endotracheal intubation and surgical measures to stabilize respiration or circulation as defined by Røise et al [12] (Table 1). Only procedures indicated by physiologic compromise were included. Thus, e.g. external pelvic fixation in the absence of severe bleeding was not counted as an emergency procedure.

**Table 1:** Emergency procedures for 142 out of a total of 441 included trauma patients admitted at the University Hospital of North Norway Tromsø during 2006-2007

Procedure	Number of patients receiving the procedure
Endotracheal intubation (percentage of total)	98 (22%)
Chest tube insertion (percentage of total)	60 (14%)
Hemostatic surgery in the abdomen (percentage of total)	10 (2.3%)
Hemostatic surgery in the pelvis with packing (percentage of total)	1 (0.2%)
Thoracotomy (percentage of total)	5 (1.1%)
Primary stabilization of fractures with external fixation (percentage of total)	22 (5.0%)

### Evaluation

The TTA protocol was evaluated against two triage standards, i.e. either ISS > 15 or an emergency procedure performed. The calculations are described in Table 2. Overtriage is defined as the fraction of TTA where the patients are not severely injured (ISS ≤15) or did not undergo an emergency procedure. Undertriage is defined as the fraction of patients admitted without TTA despite severe injuries (ISS>15) or receiving an emergency procedure.

**Table 2:** Performance of the trauma team activation protocol during 2006-2007 at the University Hospital of North Norway Tromsø, evaluated with injury severity (ISS>15) and need for emergency procedure, n = 441

2 × 2 table for calculations on performance by injury severity				2 × 2 table for calculations on performance by need for emergency procedure			
	**ISS > 15**	**ISS ≤ 15**	**Sum**		**Procedure**	**No procedure**	**Sum**

TTA	110 (a)	272 (b)	382	TTA	112 (a)	270 (b)	382
No TTA	51 (c)	unknown (d)	n/a	No TTA	30 (c)	unknown (d)	n/a

Sum	161	n/a	n/a	Sum	142	n/a	n/a

Performance by injury severity				Performance by need for emergency procedure			

Sensitivity	PPV	Overtriage	Undertriage	Sensitivity	PPV	Over Triage	Undertriage
68%	29%	71%	32%	79%	29%	71%	21%

Sensitivity = a/(a + c), specificity = d/(b + d), Positive Predictive Value (PPV) = a/(a + b), Negative Predictive Value (NPV) = d/(c + d), Overtriage = 1 - PPV = b/(a + b), Undertriage = 1 - Sensitivity = c/(a + c). N/a = not applicable.

Specificity and NPV are not applicable to the dataset, as (d) is unknown because the total number of minor injuries can not be identified.

The ability of a criterion to predict severe trauma and/or need for an emergency procedure is given as the proportion of patients that fulfilled the specific criterion that also had an ISS >15 and/or were subject to an emergency procedure. SPSS 16.0 (Chicago, Illinois, USA) was used for all analyses.

### Ethics

The study was approved by the Norwegian Data Inspectorate and the Regional Committee for Medical Research Ethics.

## Results

### Main characteristics of the material

A total of 441 patients were included, of whom 382 were received by the trauma team. Most were males (72%), blunt injuries dominated (98%) and the median ISS was 9. The main characteristics of the study population are given in Table 3.

**Table 3:** Main characteristics of the injured patients admitted at the University Hospital of North Norway Tromsø, n = 441

Male patients (percentage of total)	317 (72%)
Median age in years (interquartile range)	28 (19-50)
Median ISS (interquartile range)	9 (1-18)
30 day mortality, patients (percentage of total)	29 (6.6%)
Penetrating injuries, patients (percentage of total)	10 (2%)
Blunt injuries, patients (percentage of total)	431 (98%)
Interhospital transfer, patients (percentage of total)	90 (20%)

All patients admitted with a trauma team and all patients with Injury Severity Score (ISS) > 15 or receiving an emergency procedure (for stabilization of compromised airways, respiration or circulation) during 2006-7

Documentation of the basis for TTA was missing in 26 cases. Thus, the criteria applied for TTA was found in 356 (93%) of the patient records.

### Evaluation of the TTA protocol

The overall performance of the TTA protocol is described in Table 2. With the occurrence of severe injuries (ISS>15) as the standard of reference, the overtriage was 71% and undertriage 32%. When evaluated against the need for emergency procedures, the over- and undertriage was 71% and 21%, respectively.

The individual criteria were assessed separately (Table 4). Those of the vital functions category performed well, as more than half of the patients fulfilling any single criterion had ISS > 15 and/or underwent an emergency procedure. Fulfillment of extent-of-injury criteria was sparsely recorded. MOI observations were commonly reported. Patients who fulfilled some of the MOI-criteria only, were rarely severely wounded or undergoing emergency procedures (Table 5).

**Table 4:** An analysis of individual criteria applied for trauma team activation

Criteria category	Criterion	Criterion applied to the patient (no. of patients)	Criterion applied to a severely injured patient (ISS>15), (no. of patients)	Criterion applied to a patient receiving an emergency procedure (no. of patients)
Vital functions	1. Airway obstruction, stridor	2	2 (100%)	0
	2. Tachypnoe (adults, respiratory rate>30)	11	7 (64%)	6 (55%)
	3. Respiratory rate <10	15	11 (73%)	12 (80%)
	4. Heart rate>130 (adults)	5	4 (80%)	4 (80%)
	5. Systolic BP <90 mmHg	26	23 (88%)	21 (81%)
	6. Lowered level of consciousness (GCS <13) > 5 min	114	65 (57%)	65 (57%)
	7. Convulsions	1	0	0
	8. Dilated or not responding pupils	15	12 (80%)	13 (87%)

Extent of injuries	9. Flail chest	6	3 (50%)	4 (67%)
	10. Unstable fracture of the pelvis	7	5 (71%)	5 (71%)
	11. Fracture in two or more long bones	2	1 (50%)	1 (50%)
	12. Traumatic amputation or crush injury above wrist/ankle	3	3 (100%)	3 (100%)
	13. Injury in two or more body regions (head/neck/chest/abdomen/pelvis/femur/back)	121	46 (38%)	46 (38%)
	14. Paralysis	14	10 (71%)	6 (43%)
	15. Penetrating injury of the head/neck/chest/abdomen/pelvis/groin/back)	6	3 (50%)	4 (67%)
	16. 2. or 3. degree burn injury>15% body surface (children>10%)	1	1 (100%)	1 (100%)
	17. Burn injury with inhalation injury	1	1 (100%)	1 (100%)
	18. Hypothermia (core temperature <32°C)	6	4 (67%)	5 (83%)

Mechanism of injury	19. Ejected from vehicle	9	4 (44%)	4 (44%)
	20. Co passenger dead	6	6 (100%)	5 (83%)
	21. Trapped in wreck	24	11 (46%)	11 (46%)
	22. Pedestrian or cyclist hit by motor vehicle	38	14 (37%)	14 (37%)
	23. Motorcycle accident	37	6 (16%)	7 (19%)
	24. Considerable deformation of vehicle passenger compartment	71	12 (17%)	12 (17%)
	25. Traffic accident with speed>60 km/h	137	26 (19%)	25 (18%)
	26. Fall from >5 m	28	15 (54%)	13 (46%)
	27. Avalanche accident	8	5 (63%)	3 (38%)

ISS: Injury Severity Score, ISS > 15: Severely injured patient, GCS: Glasgow Coma Score

Emergency procedure: endotracheal intubation, chest tube insertion, hemostatic surgery in the abdomen or pelvis, thoracotomy or fracture stabilization

Individual criteria applied for trauma team activation based on prehospital information in potentially severely injured patients admitted at the University Hospital of North Norway Tromsø during 2006-7, n = 382. Each patient may have more than one criterion applied.

**Table 5:** Potentially severely injured patients admitted with trauma team activation and with one or more criteria applied in the mechanism-of-injury group, and no criteria applied in vital-functions or extent-of-injury group, n = 132

Criterion		Criterion applied to the patient (no. of patients)	Criterion applied to a severely injured patient (ISS>15), (no. of patients)	Criterion applied to a patient receiving an emergency procedure (no. of patients)
Mechanism of injury	19. Ejected from vehicle	2	0	0
	20. Co passenger dead	0	0	0
	21. Trapped in wreck	6	2 (33%)	4 (67%)
	22. Pedestrian or cyclist hit by motor vehicle	17	3 (18%)	2 (12%)
	23. Motorcycle accident	14	0	1 (7%)
	24. Considerable deformation of vehicle passenger compartment	41	2 (5%)	3 (7%)
	25. Traffic accident with speed>60 km/h	67	3 (4%)	6 (9%)
	26. Fall from >5 m	7	3 (43%)	3 (43%)
	27. Avalanche accident	4	1 (25%)	0

ISS: Injury Severity Score, ISS > 15: Severely injured patient, GCS: Glasgow Coma Score

Emergency procedure: endotracheal intubation, chest tube insertion, hemostatic surgery in the abdomen or pelvis, thoracotomy or fracture stabilization. Each patient may have more than one criterion applied.

Endotracheal intubation was the most common emergency procedure and was performed in almost one in four patients received by the trauma team (Table 1). Among emergency surgical procedures, chest tube insertion and external fracture stabilization dominated. Less than 3% of all injured patients were emergently operated by laparotomy or thoracotomy for stabilization of respiration or circulation (Table 1).

### Undertriaged patients

Undertriage was 32% evaluated against the frequency of severe injuries (ISS>15) and 21% evaluated against the occurrence of emergency procedures (Table 2). Two thirds of those undertriaged had head injuries only and/or were transferred from another hospital (Table 6).

**Table 6:** Undertriaged patients, n = 59

	Number of patients
Male patients (percentage of total)	47 (80%)
Median age (interquartile range)	57 years (38-70)
Median ISS (interquartile range)	16 (16-24)
30 day mortality (percentage of total)	4 (7%)
Transfer from a local hospital (percentage of total)	35 (59%)
Transfer from a local hospital with isolated head/neck-injury (percentage of total)	26 (44%)
Admitted directly in UNN Tromsø with isolated head/neck injury (percentage of total)	10 (17%)
Intubated before transfer to UNN (percentage of total)	18 (31%)
Intubated after arrival at UNN (percentage of total)	2 (3%)
Emergency surgery at local hospital (procedure)	1 (chest tube)
Emergency surgery after arrival at UNN (procedure)	9 (chest tubes only)

Severely injured patients (Injury Severity Score>15) or patients receiving an emergency procedure (for stabilization of compromised airways, respiration or circulation) admitted without trauma team activation at the University Hospital of North Norway Tromsø during 2006-7.

### Mechanism-of-injury criteria for TTA

The lower predictive value of the MOI criteria prompted us to investigate the potential performance of the TTA protocol without MOI criteria. We found that for 14 patients with ISS>15, the trauma team was activated on the basis of MOI criteria alone. Five of these patients were transferred from another hospital. These 14 had a mean ISS of 26, a mean age of 34 years, 10 were male, and one patient died within 30 days. Five were endotracheally intubated and six underwent at least one emergency surgical procedure.

For these 14 patients, the following MOI criteria were used to activate the trauma team (number of patients in parentheses): 21. Trapped in wreck (2), 22. Pedestrian or cyclist hit by motor vehicle (3), 24. Considerable deformation of vehicle passenger compartment (2), 25. Traffic accident with speed>60 km/h (3), 26. Fall from >5 m (3) and 27. Avalanche (1).

## Discussion

The precision of TTA criteria is important both for ensuring adequate therapeutic steps for the severely injured as well as for hospital resource utilization. Any TTA protocol should be validated to fit the local case load and trauma pattern.

The present study reveals that the patient records' documentation of the basis for TTA should have been better. Measures to improve this are necessary for continuous system surveillance. In addition, not every criterion is evaluated for each patient; emphasis is apparently laid on vital functions and mechanism of injury. An extent of injury-criterion requires an extensive clinical examination by the prehospital personnel. The first report to the EMDC is often given before this has been accomplished. For this reason, performance parameters in the extent-of-injury-group must be considered with caution.

Ideally, the criteria applied for activating the trauma team should be recorded prospectively [16]. Instead, our study may be biased by some extent of under-reporting. However, to include all available information at the time of TTA, the trauma team's admission note in the patient record was added to the EMDC data for completeness. We believe that we thus have been able to reveal practically all clinical data known to the EMDC prior to admission.

Before the UNN TTA protocol was made mandatory in 2004, overtriage was 58% and undertriage was 50% [9]. At that time, TTA was decided by the trauma leader's assessment alone, based on available prehospital information from the EMDC and a recommended, though not mandatory, set of criteria. The present protocol has successfully reduced the undertriage, but at the cost of an increased overtriage.

We report that MOI TTA criteria have a lower predictive value than those based on extent of injury or physiological compromise. This is consistent with the results from earlier studies [5-7]. If our findings were to elicit a change to our clinical decision rules, a prospective validation of the revised criteria would be preferable. The local trauma pattern may differ considerably between services, and this affects the applicability of revised TTA criteria in other patient populations than the one they were derived in [17]. However, we believe the data we present may be used for a revision of the TTA criteria in our own university hospital setting.

Undertriage mainly affected patients transferred from local hospitals and patients with head injuries (Table 6). In our hospital the same TTA criteria apply for interhospital transfers as for patients admitted directly from the scene of injury. Thus, transferred patients are included in our analysis. We have previously shown that many trauma patients transferred in the acute phase are not adequately diagnosed and stabilized [18]. Minimization of undertriage requires decision-makers to strictly comply with our TTA protocol in all trauma patients.

There have been various approaches to minimize overtriage. In two Scandinavian level I Trauma centers (Aarhus hospital, Aarhus, Denmark and St. Olav's University Hospital, Trondheim, Norway) MOI was shown to be the set of criteria with lowest performance, which is in line with our findings. Particularly the vehicle speed criterion was shown to be inaccurate [5,7].

Also in a study from Viborg, Denmark, the MOI criteria was shown to have low positive predictive value. In a revised version of the Viborg TTA protocol, fulfillment of a single MOI criterion would not alone lead to TTA [6]. Based on the results of the present study, a complete removal of the MOI criteria could have left up to 14 (8.7%) out of 161 patients with ISS>15 without TTA. Almost half of these patients also required an emergency procedure. However, criterion nr 23 (Motorcycle accident), 24 (Considerable deformation of vehicle compartment) and 25 (Traffic accident with speed>60 km/h) have limited capacity to identify the seriously injured in this material. When one of these is the only criterion fulfilled, only five (3.1%) severely injured patients out of 161 with ISS > 15 and four (2.8%) out of 142 in need of emergency procedures would be identified. Criterion nr 7 (Convulsions) was only used once in a patients not severely injured. All severely injured patients fulfilling criterion nr 8 (Dilated or not responding pupils) were also identified by criterion nr 6 (Lowered level of consciousness (GCS <13) more than 5 min). For reasons stated, we suggest removing criteria nr 7, 8, 23, 24 and 25.

Criterion nr 10 (Unstable fracture of the pelvis) could be misleading. The pelvis of a patient with a suspected pelvic fracture should not be strained outside the hospital, as this may provoke bleeding. The stability of a fractured pelvis can also be difficult to assess clinically [19], and also for this reason the term "unstable" is of limited value for triage. We therefore suggest to merge pelvic fracture with criterion nr 11 (Fracture in two or more long bones), to maintain the possibility of identifying patients with potentially severely bleeding pelvic fractures.

On the basis of the presented results, we propose a revised TTA protocol (Table 7). If applied to the material studied here, the number of patients admitted with TTA would decrease by 94(25%), of whom five would have ISS >15 and four received an emergency procedure. Accordingly, overtriage would decrease from 71% to 62% with either ISS >15 or emergency procedure as the reference standard. We believe that checking every criterion on all patients, including those transferred from another hospital and those with head injuries, would compensate for the potential increase in undertriage after removal of three MOI criteria. We also believe that the revised protocol will increase the focus on physiologic and anatomical criteria, and decrease the focus on MOI criteria, which also might contribute to improve triage.

**Table 7:** The new revised criteria for activation of the trauma team at the University Hospital of North Norway Tromsø

Criteria category	Criterion
Vital functions	1. Airway obstruction, stridor
	2. Respiratory rate <10 or >30 (adults)
	3. Heart rate >130 (adults)
	4. Systolic BP <90 mmHg
	5. Lowered level of consciousness (GCS <13) >5 min

Extent of injuries	6. Flail chest
	7. Pelvic fracture. Fracture in two or more long bones
	8. Traumatic amputation or crush injury above wrist/ankle
	9. Injury in two or more body regions (head/neck/chest/abdomen/pelvis/femur/back)
	10. Paralysis
	11. Penetrating injury of the head/neck/chest/abdomen/pelvis/groin/back
	12. 2. or 3. degree burn injury >15% body surface (children >10%)
	13. Burn injury with inhalation injury
	14. Hypothermia (core temperature <32°C)

Mechanism of	15. Ejected from vehicle
injury	16. Co passenger dead
	17. Trapped in wreck
	18. Pedestrian or cyclist hit by motor vehicle
	19. Fall from >5 m
	20. Avalanche accident

Our findings are consistent with the results from similar Scandinavian studies. We advocate a more limited use of MOI criteria in our hospital, and suggest that those criteria with the lowest predictive value and highest contribution to overtriage are removed. Given these modifications, we believe that the revised protocol will reduce overtriage without any substantial increase in undertriage. The revised protocol is implemented in our hospital.

## Competing interests

The authors declare that they have no competing interests.

## Authors' contributions

TD conceived the study idea, designed the study, recorded and analysed data, and drafted the manuscript. KF contributed to recording and analysing data, and drafting the manuscript. SAO recorded data. KB contributed to the study design, data-analysis and drafting the manuscript. All authors read and approved the final manuscript.
